# Balancing Cell Growth and Product Synthesis for Efficient Microbial Cell Factories

**DOI:** 10.1002/advs.202510649

**Published:** 2025-09-18

**Authors:** Linxia Liu, Dongqin Ding, Huiying Wang, Xinyi Ren, Sang Yup Lee, Dawei Zhang

**Affiliations:** ^1^ Tianjin Institute of Industrial Biotechnology Chinese Academy of Sciences Tianjin 300308 China; ^2^ National Technology Innovation Center of Synthetic Biology Tianjin 300308 China; ^3^ University of Chinese Academy of Sciences Beijing 101408 China; ^4^ State Key Laboratory of Engineering Biology for Low‐Carbon Manufacturing, Tianjin Institute of Industrial Biotechnology Chinese Academy of Sciences Tianjin 300308 China; ^5^ School of Biological Engineering Tianjin University of Science and Technology Tianjin 300457 China; ^6^ Department of Chemical and Biomolecular Engineering (BK21 four program) Korea Advanced Institute of Science and Technology (KAIST) Daejeon 34141 Republic of Korea

**Keywords:** cell growth, dynamic regulation, fermentation process control, microbial cell factories, microbial consortia engineering, orthogonal system design, product synthesis

## Abstract

The sustainable, bio‐based production of industrially valuable chemicals and materials from renewable, non‐edible biomass through biorefineries has emerged as a vital strategy for tackling urgent global challenges, including climate change, and for realizing the “net zero carbon” commitments recently pledged by nations worldwide. Metabolic engineering has played a central role in enabling the development of microbial strains capable of efficiently overproducing a diverse array of target compounds. Nevertheless, engineered microbial cell factories often face inherent trade‐offs between product synthesis and cell growth, frequently resulting in diminished fitness or loss‐of‐function phenotypes. This review highlights recent advances in metabolic engineering strategies aims at reconciling this conflict, encompassing pathway optimization, dynamic regulation, orthogonal system design, microbial consortia engineering, fermentation process control, and integrative metabolic modeling. It also explores the remaining challenges and future directions for reprogramming microbial metabolism to harmonize growth with high‐level production.

## Graphical abstract

This review presents state‐of‐the‐art metabolic engineering strategies to balance microbial cell growth and product synthesis in biorefineries. It surveys pathway engineering, dynamic genetic circuits, orthogonal control systems, synthetic microbial consortia, and fermentation optimization, alongside integrative modeling approaches. Current challenges and future directions for reprogramming cellular metabolism to harmonize high‐level production with robust growth are also explored.

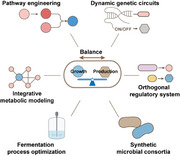

## 1 Introduction

Metabolic engineering aims to optimize the production of valuable pharmaceuticals, functional nutraceuticals, and fine chemicals by strategically redirecting metabolic flux through the rewiring and optimization of cellular metabolic networks.^[^ ^1^, ^2^ ^]^ Achieving the highest possible titer, rate (productivity), and yield is of paramount importance for developing commercially viable strains and bioprocesses.^[^ ^3^, ^4^ ^]^ Cells have naturally evolved metabolic, regulatory, and signaling systems to optimize resource utilization and maximize growth, ensuring survival and adaptability over evolutionary time.^[^ ^5^, ^6^ ^]^ However, most strategies aimed at improving product yield often deplete metabolites needed for biomass synthesis, leading to a trade‐off between cell growth and product formation.^[^ ^7^, ^8^, ^9^ ^]^ Impaired growth consequently results in reduced volumetric productivities and increased capital costs.^[^ ^10^, ^11^ ^]^ Thus, balancing cell growth with product synthesis is a critical challenge in metabolic engineering. This dynamic interplay directly impacts the overall productivity and economic viability of bioprocesses.^[^ ^12^ ^]^

**Figure 1:**
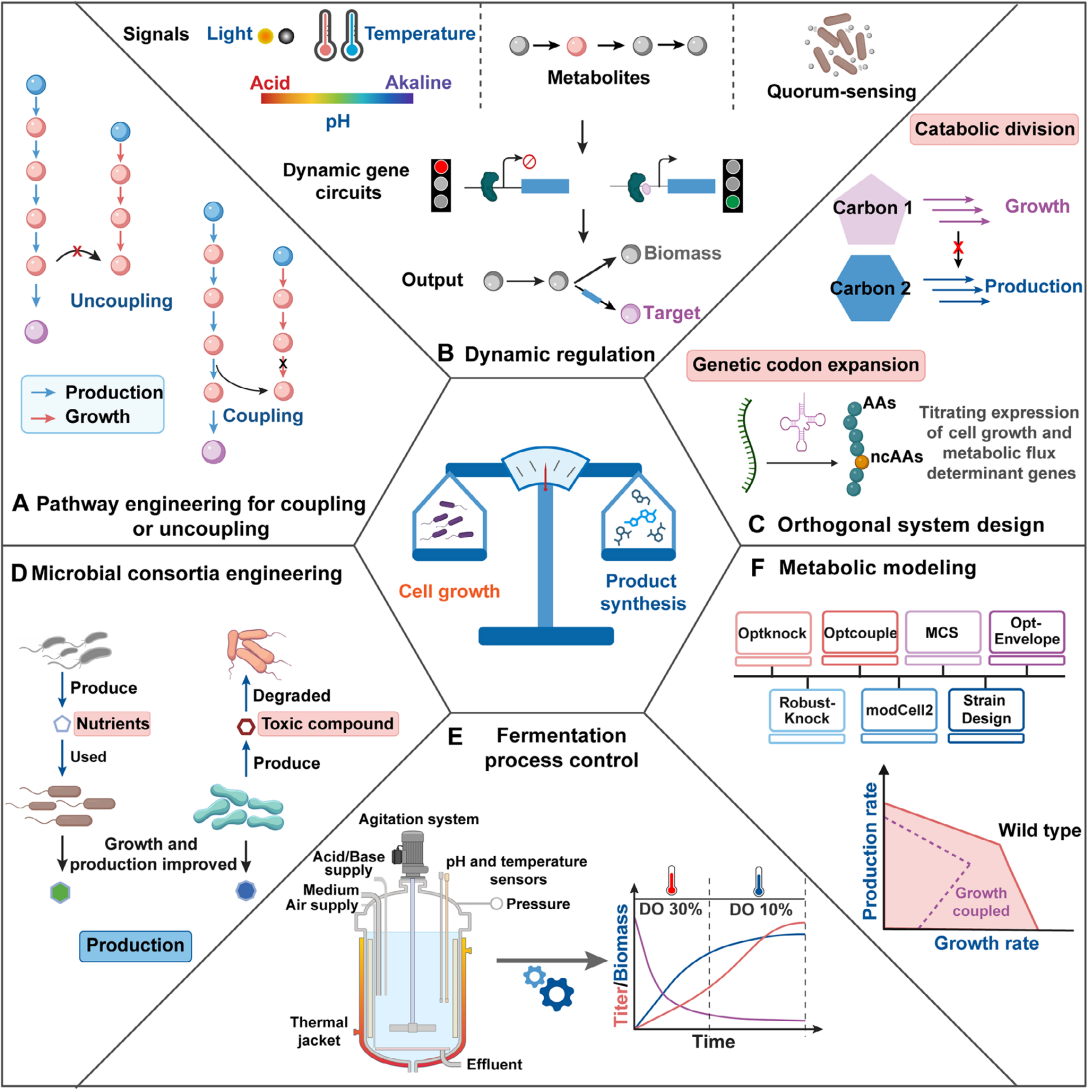
Schematic overview of strategies used to balance cell growth and product synthesis. A) Pathway engineering for coupling or uncoupling growth and product formation. The red cross indicates the knock out of the biosynthetic genes. B) Dynamic regulation using environmental circuits (light, temperature and pH), intracellular circuits, and extracellular circuits (quorum‐sensing). C) Orthogonal design to insulate cell growth and product synthesis by catabolic division of mixed carbon sources and genetic codon expansion. D) Microbial co‐culturing consortium to regulate growth and production by provide nutrients or eliminate toxic compound. E) Fermentation process control to balance biomass formation and production by regulation of dissolved oxygen (DO), temperature, etc. F) Metabolic modeling to harmonize cell growth and product synthesis using different platforms or Python. Created by Biorender and Adobe Illustrator 2024.

Cell growth plays a fundamental role in bioproduction, as it determines biomass concentration and, by extension, the number of active cell factories per unit volume, ultimately influencing the overall process productivity.^[^ ^13^ ^]^ Robust cell growth requires substantial allocation of cellular energy and resources for the synthesis of proteins, lipids, nucleic acids, and other cellular components.^[^ ^14^, ^15^ ^]^ While strong growth is essential for establishing a productive cell factory, excessive diversion of resources toward biomass accumulation can compromise the biosynthesis of target products.^[^ ^16^ ^]^ This arises because core metabolic pathways are naturally tuned to support growth, forcing primary and secondary metabolites of interest to compete for limited cellular resources.^[^ ^17^, ^18^, ^19^ ^]^ The key challenge, therefore, is to redirect or enhance metabolic flux toward product synthesis while maintaining sufficient flux for essential growth processes. Striking this balance is delicate: overemphasis on product synthesis can result in insufficient biomass, reducing both productivity and yield. Given the complexity and dynamic nature of the growth‐production relationship, a range of strategies has been developed to optimize this balance, including orthogonal design, dynamic regulation, and fermentation control process, among others (**Figure** **1**).^[^ ^20^, ^21^, ^22^, ^23^ ^]^ Orthogonal design seeks to decouple growth and production processes, allowing them to occur simultaneously without cross‐interference. This includes approaches such as parallel pathway engineering, carbon source partitioning, and codon expansion.^[^ ^24^ ^]^ Dynamic regulation involves the temporal control of substrate utilization or enzymatic activities, enabling shifts between growth and production phases in response to cellular or environmental cues.^[^ ^17^ ^]^ Meanwhile, precise control of fermentation parameters at the industrial scale can fine‐tune regulatory circuits to direct metabolic flux toward product formation.^[^ ^25^, ^26^ ^]^ These approaches are unified under the framework of systems metabolic engineering, which combines metabolic engineering with systems biology and synthetic biology to design sophisticated control mechanisms. This enables temporal separation of growth and production phases or flux redirection in response to intracellular states and environmental signals.^[^ ^27^, ^28^, ^29^ ^]^ Understanding and managing the balance between cell growth and product synthesis is essential for the development of efficient, high‐yield, and sustainable bioprocesses. While achieving precise control over this balance remains a major challenge, it also presents significant opportunities for advancing the fields of biotechnology and industrial microbiology.

This review focuses on techniques and strategies for balancing cell growth with product synthesis, illustrated through recent examples of successful metabolic engineering. We provide guidance on selecting appropriate approaches and understanding their limitations. Finally, we discuss the current challenges in this field and highlight the potential of systems metabolic engineering as a key strategy for the successful development of microbial cell factories.

## 2 Pathway Engineering for Coupling or Uncoupling Growth and Product Formation

In synthetic biological pathways, targeted metabolic flux must shift from endogenous metabolism, which primarily supports cell growth, toward bioproduction–a transition that often conflicts with the cell's survival needs due to competition for shared precursors and energy between growth and product synthesis.^[^ ^24^, ^30^ ^]^ Optimizing the flow of metabolic intermediates between these competing pathways is therefore critical for improving overall productivity.^[^ ^31^ ^]^ A well‐balanced flux ensures that neither growth nor production is disproportionately prioritized at the expense of the other. Consequently, innovative synthetic pathway design is essential for constructing efficient microbial factories capable of producing target compounds (Figure 1A).

**Figure 2:**
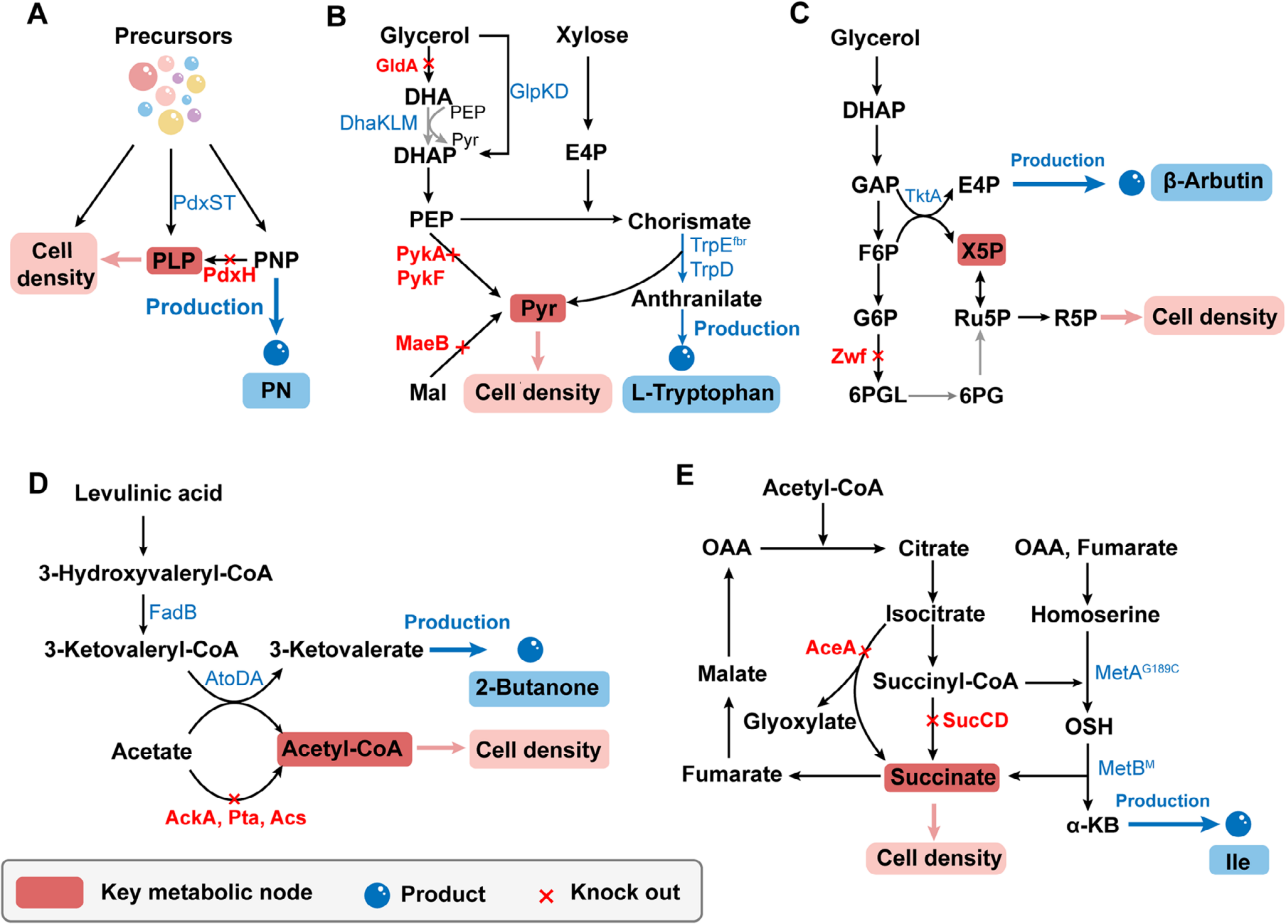
Pathway engineering has been used to either decouple or couple microbial production to balance cell growth and product synthesis. A) Growth‐decoupled production of PN by deletion of *pdxH* and introduction of heterologous *pdxST* in *E. coli*. Adapted with permission.^[^ ^32^ ^]^ Copyright 2023, Springer Nature. B) A pyruvate‐driven metabolic scenario for growth‐coupled microbial production. Adapted with permission.^[^ ^33^ ^]^ Copyright 2019, Elsevier. C) A growth‐coupled mechanism for high‐yield production of β‐arbutin from glycerol in *E. coli*. Adapted with permission.^[^ ^34^ ^]^ Copyright 2023, Elsevier. D) Growth‐coupled bioconversion of levulinic acid to butanone based on the metabolic node acetyl‐CoA by preventing native acetate incorporation (Δ*ackA*, Δ*pta*, Δ*acs*). Adapted with permission.^[^ ^35^ ^]^ Copyright 2019, Elsevier. E) Overview of the coupling between TCA cycle and L‐isoleucine biosynthesis. Adapted with permission.^[^ ^36^ ^]^ Copyright 2024, Elsevier. Red font shows the deletion of the key enzymes. The metabolites in the red boxes represent key nodes for coupling or uncoupling. Red crosses indicate the disruption of metabolic pathways. The gray solid lines indicate reaction blockages due to gene knockouts. PNP, pyridoxine 5′‐phosphate; PLP, pyridoxal 5′‐phosphate; PN, pyridoxine; GAP, glycerol 3‐phosphate; DHAP, glycerone phosphate; PEP, phosphoenolpyruvate; PYR, pyruvate; G6P, glucose 6‐phosphate; F6P, Fructose‐6‐phosphate; 6PGL, 6‐Phosphogluconolactone; 6PG, 6‐Phosphogluconic acid; X5P, Xylulose 5‐phosphate; Ru5P, Ribulose 5‐phosphate; R5P, Ribose 5‐phosphate; E4P, Erythrose 4‐phosphate; Mal, malate; OAA, Oxaloacetate; OSH, O‐succinyl‐L‐homoserine; Ile, L‐isoleucine; α‐KB, α‐ketobutyrate. PdxH, PNP oxidase; PdxST, PLP synthase PdxS and glutamine aminotransferase PdxT; Zwf, glucose 6‐phosphate dehydrogenase; PykAF, pyruvate kinases; TktA, transketolase I; GldA, glycerol dehydrogenase; DhaKLM, phosphoenolpyruvate (PEP)‐dependent dihydroxyacetone kinase; TrpED, anthranilate synthase; MaeB, malate dehydrogenase; FadB, 3‐hydroxyacyl‐CoA dehydrogenase; AtoDA, acetoacetyl‐CoA transferase; AckA, acetate kinase; Pta, phosphate acetyltransferase; Acs, acetyl‐CoA synthetase. AceA, isocitrate lyase; MetA^G189C^, feedback‐resistant homoserine O‐succinyltransferase; MetB^M^, cystathionine γ‐synthase mutant.

In a recent study, *Escherichia coli* was engineered to produce vitamin B_6_ by decoupling pyridoxine (PN) production from cell growth.^[^ ^32^ ^]^ This was accomplished by establishing a parallel metabolic pathway linked to growth through the cofactor pyridoxal 5′‐phosphate (PLP). The native *pdxH* gene, which encodes PNP oxidase responsible for PLP production, was replaced by the *pdxST* genes from *Bacillus subtilis*, which encode enzymes that directly synthesize PLP. The resulting optimized strain enabled a parallel pathway for the de novo biosynthesis of vitamin B_6_, via the direct synthesis of B_6_ vitamers. This strain enhanced PN production by redirecting metabolic flux from PNP toward PN instead of PLP (**Figure** **2**A).

Alternatively, by manipulating and rewiring microbial metabolism, the synthesis of target compounds can be made essential for cell growth, resulting in a “growth‐driven” phenotype that enhances cellular robustness. Growth coupling imposes a selective pressure for production by aligning cellular survival with product formation, thereby improving strain adaptability and increasing fermentation productivity.^[^ ^37^, ^38^ ^]^

Pyruvate, a central metabolite linking glycolysis and the TCA cycle,^[^ ^39^, ^40^ ^]^ has been employed as a metabolic node for growth‐coupling strategies. In one study, a pyruvate‐driven system was constructed by rewiring carbon metabolism and disrupting endogenous pyruvate‐generating pathways to enforce synthetic, pyruvate‐forming routes, thereby ensuring pyruvate regeneration for growth.^[^ ^33^ ^]^ Anthranilate (AA) was selected as the target compound due to its biosynthetic pathway releasing pyruvate. Disruption of key pyruvate‐producing genes‐*pykA*, *pykF*, *gldA* and *maeB*‐significantly impaired cell growth in glycerol minimal medium, likely due to insufficient pyruvate supply. Overexpression of a feedback‐resistant anthranilate synthase (TrpE^fbr^G) on a plasmid in engineered *E. coli* strains restored growth and enhanced AA production (Figure 2B). This pyruvate‐driven strategy resulted in over a 2‐fold increase in the production of AA and its derivatives, including L‐tryptophan (L‐Trp) and *cis*, *cis*‐muconic acid (MA).^[^ ^33^ ^]^

Erythrose 4‐phosphate (E4P), a key metabolite linking the pentose phosphate pathway (PPP) and glycolysis,^[^ ^41^ ^]^ was similarly employed. Along with xylulose 5‐phosphate (X5P), E4P can be converted into fructose‐6‐phosphate (F6P) and glyceraldehyde‐3‐phosphate (GAP) via the reverse reactions catalyzed by transketolase (TktA) and transaldolase (Tal).^[^ ^34^ ^]^ To drive this reverse flux, the carbon flow through the PPP was blocked by deleting *zwf*.^[^ ^34^ ^]^ X5P was subsequently converted into ribose‐5‐phosphate (R5P) through reversible reactions catalyzed by ribulose‐phosphate 3‐epimerase (Rpe) and ribose‐5‐phosphate isomerase (Rpi) (Figure 2C).^[^ ^42^ ^]^ Because R5P is essential for nucleotide biosynthesis, coupling E4P formation with R5P biosynthesis effectively linked target compound production to growth. Using this strategy, efficient microbial synthesis of β‐arbutin was achieved, reaching titers of 7.91 g L^−1^ in shake flasks and 28.1 g L^−1^ in fed‐batch fermentation.^[^ ^34^ ^]^

Theoretically, by analyzing metabolic state transitions between growth and production phases, it is possible to couple product synthesis with biomass formation via any of the 12 central precursor metabolites: glucose 6‐phosphate, fructose 6‐phosphate, GAP, 3‐phosphoglycerate, phosphoenolpyruvate, pyruvate, acetyl‐CoA, α‐ketoglutarate, succinyl‐CoA, oxaloacetate, R5P, and E4P.^[^ ^43^ ^]^ These precursors lie at metabolic branch points and serve as the foundation for biosynthesis of amino acids, nucleotides, and other macromolecule.^[^ ^44^ ^]^ For example, acetyl‐CoA‐mediated growth coupling was used to produce butanone (Figure 2D). Native acetate assimilation pathways (AckA, Pta, Acs) were deleted, and complete levulinic acid (LA) catabolism was blocked by removing key thiolases (FadA, FadI, AtoB). The only available route to acetyl‐CoA was through CoA transfer from 3‐hydroxyvaleryl‐CoA to acetate, effectively coupling acetate assimilation to butanone synthesis. This strategy yielded a butanone titer of 855 mg L^−1^ and completely consumed the supplied acetate.^[^ ^35^ ^]^ Similarly, a succinate‐driven growth‐coupling strategy was employed to enhance L‐isoleucine production (Figure 2E).^[^ ^36^ ^]^ Deletion of *sucCD* and *aceA* blocked succinate formation via the TCA and glyoxylate cycles, respectively. Overexpression of MetA^G189C^ and MetB^M^ enabled an alternative L‐isoleucine biosynthetic route that bridged the resulting metabolic gap (Figure 2E).^[^ ^36^ ^]^ In addition, non‐central precursor metabolites can also be used to construct coupled designs that link cellular growth to product formation. Accumulation of propionyl‐CoA and methylmalonyl‐CoA in *Corynebacterium glutamicum* inhibits growth, but introducing a methylmalonyl‐CoA–dependent polyketide synthase (germicidin synthase) relieves this inhibition, effectively coupling growth to polyketide production. Leveraging this coupling via adaptive laboratory evolution led to improved germicidin titers and mutation‐driven metabolic rewiring.^[^ ^45^ ^]^

Establishing a growth‐driven phenotype often requires routing the biosynthesis of one or more essential growth components through the synthetic pathway associated with the target compound. However, this approach can limit adaptability. Reconstituted gene circuits frequently require extensive trial‐and‐error design optimization and are vulnerable to disruption by the complex intracellular environment. A practical compromise is controlled downregulation of competing pathways to redirect flux without completely suppressing biomass formation. For instance, downregulating *pgi* (encoding glucose‐6‐phosphate isomerase) by replacing its start codon ATG with GTG redirected carbon flux toward the PPP, increasing NADPH availability for L‐arginine biosynthesis while preserving enough glycolytic flux to support cell growth.^[^ ^46^ ^]^

Pathway bypass strategies also offer effective means to decouple production from growth. Branched‐chain C5 alcohols can be synthesized through the mevalonate (MVA) or non‐mevalonate (MEP/DXP) pathways.^[^ ^47^, ^48^ ^]^ Both share isopentenyl pyrophosphate (IPP) as a common precursor, which hinders independent regulation of these pathways. Kang et al. (2017) engineered novel IPP‐bypass MVA pathways using promiscuous activities of phosphomevalonate decarboxylase (PMD) and the *E. coli* phosphatase AphA.^[^ ^49^ ^]^ These routes eliminated dependency on IPP, reduced energy demand, escaped native regulation, and avoided IPP toxicity, thereby decoupling product synthesis from growth. The “IPP‐bypass” strategy has also been applied to the production of other isopentenols.^[^ ^50^ ^]^

Advances in synthetic biology are enabling increasingly sophisticated regulatory designs. Dynamic control systems responsive to intracellular or extracellular signals allow real‐time adjustment of pathway activity. These may involve feedback loops, metabolite sensors, or external triggers such as light or temperature to regulate gene expression.^[^ ^51^, ^52^ ^]^ Another promising approach is orthogonal designs, which insulates synthetic pathways from the host's native regulatory network. Such systems minimize cross‐talk and enhance pathway stability and efficiency.^[^ ^30^ ^]^ Orthogonal and modular designs also support predictable behavior and facilitate iterative optimization. These strategies will be revisited in greater detail below, presenting case studies that illustrate their practical applications in developing robust, efficient microbial cell factories.

## 3 Dynamic Regulation using Metabolic Switch or Quorum Sensing Systems

The use of microorganisms to convert inexpensive raw materials into high‐value target products has become an increasingly prominent trend in industrial synthesis in recent years.^[^ ^53^, ^54^ ^]^ With continued advances in synthetic biology–alongside supporting technologies such as metabolomics and genomics–the complex metabolic pathways and regulatory networks within cells are becoming increasingly well understood.^[^ ^55^, ^56^ ^]^ However, metabolism is inherently dynamic. Following nutrient uptake, a series of enzymes and proteins continuously catalyze the conversion of raw materials into diverse metabolites and final products. As metabolism proceeds, both the intracellular and extracellular environments undergo various changes, such as the accumulation of metabolic by‐products, pH shifts due to acidic or alkaline compound build‐up, fluctuations in DO levels, and the presence of toxic intermediate metabolites.^[^ ^57^ ^]^ These changes can affect cell growth and product synthesis to varying degrees. Therefore, mitigating the adverse impacts of such metabolic fluctuations on target product formation is a critical area of investigation.^[^ ^29^ ^]^

In recent years, the use of biosensors to monitor intracellular chemical signals and extracellular environmental conditions has gained widespread attention and application.^[^ ^58^, ^59^ ^]^ By responding to metabolic fluctuations, biosensors enable autonomous and dynamic regulation of metabolic flux through synthetic pathways, offering more precise and responsive metabolic control.^[^ ^60^, ^61^ ^]^ Compared to static regulation mechanisms‐such as constitutive promoter or ribosome binding site (RBS) control‐dynamic regulation provides several notable advantages.^[^ ^62^ ^]^ It allows real‐time adjustment of metabolic flux in response to changes in intracellular metabolite concentrations and environmental signals, thereby enhancing the stability of metabolic processes (Figure 1B). Dynamic systems enable cells to adapt rapidly to complex and changing conditions, overcoming the limitations of static regulation, which often suffers from rigidity and inefficient resource allocation.^[^ ^17^ ^]^ As a result, dynamic regulation improves resource utilization, reduces by‐product formation, and increases the efficiency of target product production. Moreover, the integration of multiple input signals enables comprehensive and adaptive control of metabolic networks, paving the way for more intelligent, automated, and robust bioproduction systems.

### 3.1 Dynamic Regulation by Light, Temperature, and pH

Environmental responsive dynamic regulation optimizes cellular function within fermentation systems by continuously monitoring and adjusting environmental factors in real time using sensors and data acquisition systems.^[^ ^12^, ^17^ ^]^ This approach specifically addresses temporal fluctuations in variables such as light, temperature, and pH, enabling precise control over cell growth and metabolic processes. By maintaining optimal conditions, it ultimately enhances production efficiency and product quality. Since light can influence cell behavior in both spatial and temporal dimensions, Wu et al. (2021) developed an optogenetic‐CRISPR interference (opto‐CRISPRi) system that allocates metabolic resources according to different optical signal frequencies, enabling bacteria to toggle between the growth and production phases.^[^ ^63^ ^]^ This system dynamically suppresses central metabolism and competing pathways using the blue light‐sensitive protein EL222 to regulate expression of the dCpf1‐mediated CRISPRi system, leading to a 130% increase in muconic acid production.^[^ ^63^ ^]^ Liu et al. (2024) explored the lactose repressor protein LacI as a regulatory element and, through rational design and optoprotein engineering, incorporated the blue light‐responsive AsLOV2 domain to create two light/dark‐responsive LacI mutants: OptoLacIL and OptoLacID.^[^ ^64^ ^]^ These were used to develop two optogenetic *E. coli* expression systems: OptoE.coliLight and OptoE.coliDark, which respond to blue light and darkness, respectively. Compared to IPTG‐inducible systems, these light‐based systems offer a safe, non‐toxic, and more easily controllable alternative for regulating protein expression and metabolic flux. The OptoE.coliDark system, in which light inhibits and darkness induces gene expression, was effectively applied to control protein production and metabolic pathways in microbial cell factories. It enabled high‐efficiency expression of various enzyme, including glucose dehydrogenase, formaldehyde lyase, alkaline protease, and green fluorescent protein. In terms of metabolic flux regulation, darkness‐induced production of 1,3‐propanediol and ergothioneine surpassed IPTG‐induced levels by 110 and 60%, respectively.^[^ ^64^ ^]^

Thermosensitive genetic tools have been engineered for dynamic regulation, allowing precise control over the transition between cell growth and product synthesis phases. A robust and reversible thermosensitive bio‐switch (T‐switch), based on the temperature‐sensitive transcription factor CI^857^ and PhlF, was developed to enable stringent, bidirectional control of gene expression in *E. coli* over time and expression levels.^[^ ^65^ ^]^ Under varying temperature conditions, the T‐switch effectively regulated cell growth, generating tunable, tree ring‐like colony patterns on a macroscopic scale. On the microscopic level, it enabled morphological changes in cells ranging from spherical to rod‐shaped and fiber‐like forms‐and facilitated the ordered assembly of block biopolymers at the molecular level. Additionally, the T‐switch was used at 30 and 37 °C to dynamically control two separate biosynthetic pathways for 3HB‐CoA and 4HB‐CoA, enabling the de novo synthesis of the diblock copolymer PHB‐b‐P4HB and significantly improving its mechanical properties.^[^ ^65^ ^]^ Similarly, Harder et al. (2018) developed a synthetic genetic control system utilizing the temperature‐sensitive transcriptional regulator CI857.^[^ ^66^ ^]^ This system promoted rapid cell growth at 37 °C in the initial stage. In the subsequent production phase, the temperature was lowered to 28 °C, reducing TCA cycle flux and redirecting metabolic activity toward itaconic acid synthesis. This thermal shift ultimately increased the itaconic acid titer to 47 g L^−1^.^[^ ^66^ ^]^

Intracellular pH is a critical factor in numerous biological and biotechnological processes, including enzymatic activity and membrane transport.^[^ ^67^ ^]^ Changes in proton distribution can alter intracellular pH, potentially disrupting vital cellular functions.^[^ ^68^ ^]^ Accurate monitoring of intracellular pH is thus essential for understanding and controlling pH‐dependent physiological and metabolic processes. Rajkumar et al. (2016) engineered a set of strong synthetic promoters for *Saccharomyces cerevisiae*, inducible under acidic conditions (pH ≤ 3), by modifying transcription factor binding sites. These promoters were applied in low pH fermentation, resulting in a tenfold increase in lactic acid production compared to commonly used promoters. This validated a generalizable strategy for the iterative design of synthetic yeast promoters.^[^ ^69^ ^]^ Rupprecht et al. (2017) developed a genetically encoded FRET‐based pH biosensors toolbox, Fluorescence Biosensors for pH (FluBpH), using the FMN‐binding fluorescent protein EcFbFP as the donor. Unlike many GFP‐family proteins, EcFbFP exhibits exceptional stability under acidic conditions. The in vivo utility of FluBpH was demonstrated through ratiometric measurement of intracellular pH changes during acid stress in *E. coli*.^[^ ^70^ ^]^ Beyond sensing, pH itself can serve as a regulatory input for dynamic metabolic control. Yin et al. (2017) identified the Pgas promoter, which strongly induces gene under low pH conditions (pH ≈2.0) but exhibits minimal activity at pH levels above 5.0. Using Pgas in *Aspergillus niger*, they dynamically upregulated expression of the *cad* gene (encoding aconitate decarboxylase from *A. terreus*), boosting itaconic acid production to 4.92 g L^−1^.^[^ ^71^ ^]^

### 3.2 Dynamic Regulation by Sensing Physiological State and Intracellular Metabolites

In metabolic engineering, blocking competitive or by‐product synthesis pathways is a widely adopted strategy to improve the yield of target compounds by redirecting metabolic flux toward the desired biosynthetic route.^[^ ^72^ ^]^ However, this approach often has adverse effects on strain growth, leading to a substantial decline in production capacity during large‐scale fermentation. Detailed analyses typically reveal that such issues arise from the depletion of key metabolites or imbalances in reducing power caused by the disruption of specific pathways, ultimately impairing cellular function. Moreover, the excessive accumulation of certain intermediates can inhibit growth or result in inefficient resource utilization, further reducing production efficiency. Dynamic control strategies, which respond to the distinct physiological states or intracellular metabolites levels of individual cell, enable temporal and spatial regulation of critical metabolic genes at the single‐cell level.^[^ ^12^, ^73^ ^]^ Ding et al. (2025) developed a bifunctional dynamic control system that targets glucose uptake rate.^[^ ^74^ ^]^ By precisely regulating glucose assimilation at the source, this system optimizes metabolic flux distribution across central metabolic pathways. As a result, it ensures robust microbial growth, minimizes by‐product formation, and significantly enhances the yield of target compounds such as L‐tryptophan.^[^ ^74^ ^]^ This dynamic regulation approach effectively overcomes the limitations of traditional static modifications, offering a powerful and flexible alternative for optimizing microbial production systems.

High‐yield production processes often impose significant metabolic burdens on host cells, which can lead to the emergence and proliferation of non‐productive subpopulations during large‐scale fermentation.^[^ ^75^ ^]^ To mitigate this, one effective strategy involves coupling the synthesis of target compounds with cell growth, thereby enhancing strain robustness under fluctuating environmental conditions. Unlike previous sections that focused on static metabolic engineering approaches, this section introduces strategies for dynamically regulating and redesigning microbial metabolism, to enable the synthesis of metabolites essential for cell growth. This growth‐coupled design not only results in a growth‐driven phenotype but also improves cellular robustness.^[^ ^76^ ^]^ Various metabolic engineering strategies have been developed to dynamically couple growth with the production of specific metabolites or an enzymatic reactions by targeting key pathways or enzymes.^[^ ^13^, ^17^, ^29^ ^]^ For example, Lu et al. (2024) engineered a biosensor responsive to the pathway intermediate benzoic acid (BA) to regulate the expression of GltA* (R164L), thereby enhancing benzyl benzoate production.^[^ ^77^ ^]^ The resulting titer reached 1091.3 mg L^−1^–a nearly1.7‐fold increase compared with a static control system (**Table** **1**). Stella et al. (2021) integrated a synthetic circuit based on an Lrp transcription factor (TF)‐based biosensor upstream of two growth‐regulating genes, *pfkA* and *hisD*, in *C. glutamicum*. This system dynamically coupled cell growth to the intracellular concentration of branched‐chain amino acids. Moreover, process condition optimization prevented the enrichment of non‐productive mutants or “cheaters” (Table 1).^[^ ^78^ ^]^ Li et al. (2024) developed a whole‐cell biosensor for 3‐fucosyllactose (3‐FL) production. By introducing α‐L‐fucosidase into the host strain, 3‐FL was hydrolyzed into fucose, which served as the sole carbon source for cell growth, effectively coupling cell proliferation to 3‐FL synthesis. Using this biosensor‐guided screening, the production of 3‐FL was enhanced by 25 %, reaching 42.05 ± 1.28 g L^−1^ (Table 1).^[^ ^79^ ^]^ In another study, Zou et al. (2024) constructed a self‐regulated network in which salicylate‐a pathway intermediate‐served as the inducer molecule.^[^ ^80^ ^]^ During the early growth phase, no regulation was applied, allowing the cells to utilize substrates for biomass accumulation. Once salicylate reached a defined threshold, the major pyruvate flux was dynamically redirected by inhibiting *pykF*, shifting metabolism from growth to production mode. With all native pyruvate‐releasing pathways blocked, carbon flux was funneled into the 4‐hydroxycoumarin (4‐HC) biosynthesis pathway, while the pyruvate generated within this pathway continued to support cell growth. As a result, the cells were forced to produce 4‐HC to sustain growth (Table 1).^[^ ^80^ ^]^

**Table 1:** Applications of dynamic regulation for balancing microbial production and cell growth.

System	Metabolic Node or Target	Summary	Proof of concept	Refs.
Benzoic acid (BA) biosensor	GltA	A biosensor responsive to the biosynthetic pathway intermediate BA to control *gltA* expression	Benzyl benzoate	[77]
1‐butanol biosensor	1‐butanol	The technology of transcription factor‐based biosensors has enabled product‐dependent growth, enhanced the specific productivity of 1‐butanol, and achieved significant enrichment of the 1‐butanol production phenotype through synthetic selection.	1‐butanol	[76]
Lrp biosensor	PfkA and HisD	Integrating a synthetic circuit based on the Lrp biosensor upstream of two growth‐regulating genes (*pfkA* and *hisD*), as an evolution strategy to improve small molecule production	L‐valine	[78]
3‐FL whole‐cell biosensor	Fucose	3‐FL whole‐cell biosensor by coupling cell growth with production	3‐FL	[79]
PmarO‐MarR and I12AII14T‐MarR.	PEP	A self‐regulated network for dynamically balancing multiple precursors	4‐HC	[80]
L‐cysteine biosensor	L‐cysteine	A biosensor for engineering and screening L‐cysteine overproducers	L‐cysteine	[84]
CRUISE	L‐Val and L‐Leu	Self‐assembly‐aided CRUISE so that the cell resources were tilting toward achieving directed amino acids to higher alcohols conversion	Isobutanol and Isopentanol	[82]
CiMS	Icd, PykA, and SucCD (TCA cycle)	CiMS contained an ITA‐responding biosensor YpItcR/Pccl to dynamically regulate the expression of dCas9, which could target the desired genes with the corresponding designed sgRNAs to regulate the distributions of metabolic flux	Itaconic acid	[23]
Farnesyl pyrophosphate‐biosensor	Farnesyl pyrophosphate	By employing these promoters for dynamic regulation of metabolic pathways, the final product yield of the isoprenoid biosynthetic pathway in *E. coli* was successfully increased, the accumulation of toxic intermediates was reduced, and cell growth was improved.	Amorphadiene	[29]
L‐arginine biosensor	L‐arginine	With the assistance of a biosensor, a strain with an L‐arginine production of 132 g L^−1^ was successfully screened	L‐arginine	[85]
*p*‐Coumaroyl‐CoA biosensor	*p*‐coumaroyl‐CoA	By integrating biosensor‐based dynamic regulation of the metabolic pathway, a naringenin titer of 47.3 mg L^−1^ was achieved	*p*‐Coumaroyl‐CoA	[86]
Alkane biosensor	Alkane	By screening relevant enzymes AAR and ADO in the alkane synthesis pathway using a biosensor, the alkane yield was successfully increased 13‐fold	Alkane	[87]
*cis*, *cis*‐Muconic acid biosensor	*cis*, *cis*‐Muconic acid	A mutant strain with a 49.7% increase in *cis*, *cis*‐muconic acid production was successfully identified from the *cis*, *cis*‐muconic acid‐producing mutagenesis library using a biosensor‐based screening method	*cis*, *cis*‐Muconic acid	[88]
Anthranilic acid biosensor	Anthranilic acid	An anthranilic acid biosensor‐assisted cell selection technique was adopted to improve the microbial cell population composition	Anthranilic acid	[89]
Dynamic sensor‐regulator system (DSRS)	Biodiesel	The DSRS dynamically regulates the expression of genes involved in biodiesel production and improved the stability of strains	Biodiesel	[73]
Glycolate‐responsive biosensor	Glycolate	Systematically optimize the expression of multiple genes in the synthetic pathway based on biosensors	Glycolate	[90]
GECFINDER	Organic and amino acids	Report a method to rapidly convert enzymes into genetically encoded circularly permuted fluorescent protein‐based indicators to detect organic acids, and used biosensors to increase the production of L‐phenylalanine by 10 times	L‐phenylalanine	[91]
NeuAc riboswitch	N‐acetylneuraminate	Using N‐acetylneuraminate‐responsive RNAB screening, the titer of N‐acetylneuraminate improved by 39% compared with the original strain	N‐acetylneuraminate	[92]
L‐phenylalanine biosensor	L‐phenylalanine	Pathway evolution was performed with the assistance of TyrR‐based TFB and FACS‐based screening. This resulted in 5.79 g L^−1^ L‐phe being produced with an 80% improvement over the original strain	L‐phenylalanine	[93]
Caprolactam‐detecting genetic enzyme screening system (CL‐GESS)	Lactam	Established and engineered a lactam detecting biosensor (CL‐GESS) with high sensitivity and specificity	Identified a cyclase that converts 6‐ACA to ε‐ caprolactam	[94]
Protease‐based protein regulatory unit	Anhydrotetracycline	Constructed bifunctional switches that enable *E. coli* to accumulate or degrade target proteins when reprogramming metabolic flux	Shikimate, D‐xylonate	[95]
Pyruvate‐responsive genetic circuits	Pyruvate	Created a set of programmable and bifunctional pyruvate‐responsive genetic circuits for dynamic dual control (activation and inhibition) of central metabolism in *B. subtilis*	Glucaric acid	[39]
GABS	α‐Ketoglutarate and L‐arginine	Dynamically repress the flux of the competitive modules (TCA module and the arginine biosynthesis module) and simultaneously up‐regulate the expression of GABA synthetic modules after sufficient biomass accumulation	γ‐Aminobutyric acid	[96]
dTFS and dTNS	AroK and AroG^fbr^; PfkA and Ino1	Integrating dTFS and dTNS into a bifunctional molecular switch to uncoupled cell growth from the synthesis of shikimic acid and D‐glucaric acid	Shikimic acid and D‐glucaric acid.	[97]
A growth‐stage‐dependent molecular switch	PLP	A PLP sensor‐based negative feedback circuit, dynamically regulating the expression of the PLP synthetic genes and preventing excessive intracellular PLP accumulation	Cadaverine	[98]
A self‐induced dynamic temporal regulation cascade circuit library	Circuit response time interval	enable the expression of target genes with sequential changes at different time	Poly‐β‐hydroxybutyric acid	[99]

Synthetic product addiction, which links high‐yield production of a target metabolite to the expression of nonconditionally essential genes, can significantly extend the productive lifespan of engineered *E. coli* populations.^[^ ^81^ ^]^ In one implementation, the expression of two such genes, *folP* and *glmM*, was controlled using a mevalonic acid‐responsive biosensor.^[^ ^81^ ^]^ The resulting product‐addicted strain maintained high levels of mevalonic acid production over 95 generations‐equivalent to the number of generations required for industrial‐scale fermentations exceeding 200 m^3^. In contrast, a non‐addicted control strain ceased production entirely within the same timeframe.^[^ ^81^ ^]^ To optimize resource allocation for higher alcohol production while maintaining a stable flux of amino acids essential for normal strain growth, Chen et al. (2024) developed a concentration recognition‐based auto‐dynamic regulation system (CRUISE).^[^ ^82^ ^]^ This system integrates an amino acid‐responsive transcriptional attenuator (IvbL) and a higher alcohol‐responsive transcriptional activator (BmoR). Combined with scaffold‐based enzyme self‐assembly, CRUISE enabled the production of 40.4 g L^−1^ of isobutanol in a bioreactor (Table 1).^[^ ^82^ ^]^ CRISPR interference (CRISPRi), a powerful gene regulation technique based on a modified, RNA‐guided DNA endonuclease from the type II CRISPR/Cas9 system, has also been adapted for dynamic control.^[^ ^83^ ^]^ Zhao et al. (2022) developed a CRISPRi‐mediated self‐inducible system (CiMS), which integrates an itaconic acid (ITA)‐responsive biosensor module (YpItcR/Pccl) with a CRISPRi‐based regulatory module (Table 1).^[^ ^23^ ^]^ This system dynamically modulates TCA cycle gene expression (*icd*, *pykA*, and *sucCD*) to balance flux between growth and production, thereby enhancing ITA production capacity.^[^ ^23^ ^]^ As summarized in Table 1, these examples highlight the power of autonomous biosensors in dynamically regulating cell growth and metabolite production. Such systems effectively minimize the inherent competition between biomass formation and compound synthesis, offering a robust strategy for improving the stability and efficiency of engineered microbial production platforms.

Promoters regulate gene expression in response to internal or external signals, thereby influencing both cell growth and product synthesis.^[^ ^100^, ^101^ ^]^ The use of growth phase‐dependent promoters (GPPs) is a dynamic regulation strategy that enables phase‐specific transcriptional control of target genes.^[^ ^96^ ^]^ To further enhance dynamic regulation, degrons protein tags that promote targeted degradation can be employed at the post‐translational level to increase protein turnover and regulatory sensitivity.^[^ ^102^ ^]^ Wei et al. (2022) developed a tunable, growth phase‐dependent, autonomous bifunctional genetic switch (GABS) by coupling growth phase‐responsive promoter (P_CP_2836_) with a degron.^[^ ^96^ ^]^ This system enabled dynamic redirection of carbon flux from a growth‐focused metabolic state to a production‐focused one, resulting in high‐level GABA production from low‐cost glycerol in *C. glutamicum* (Table 1). Hou et al. (2020) designed two systems: a dynamic turn‐off switch (dTFS) and a dynamic turn‐on switch (dTNS), both leveraging growth phase‐dependent promoters and degrons (Table 1).^[^ ^97^ ^]^ By integrating these systems into a bifunctional molecular switch, they decoupled cell growth from the production of shikimic acid and D‐glucaric acid.^[^ ^97^ ^]^ This strategy led to the production of 14.33 g L^−1^ shikimic acid and achieved the highest reported D‐glucaric acid productivity at 0.0325 g L^−1^ h^−1^. In another example, replacing the native ACC1 promoter with the growth‐regulated PDC1 promoter in *S. cerevisiae* more than doubled lycopene yield, reaching 37.19 mg g^−1^ CDW‐significantly higher than that of the basal strain.^[^ ^21^ ^]^ Promoters responsive to intracellular metabolite concentrations can also be used to dynamically regulate metabolic flux between growth and production. Zhang et al. (2022) replaced the native promoters of *zwf*, *pgi*, and *pfk1*‐key genes in central carbon metabolism with a glycerol‐inducible promoter.^[^ ^103^ ^]^ By sequentially adding glycerol and glucose, the engineered strain could be switched from a growth mode to a production mode, resulting in a 4.9‐fold increase in inositol production. Furthermore, dynamically activated promoters can be harnessed to fine‐tune the production of desired metabolites based on environmental conditions. Maury et al. (2018) studied promoter dynamics under batch cultivation using glucose as the sole carbon source or under glucose‐limiting conditions.^[^ ^104^ ^]^ By expressing the 3‐hydroxypropionic acid (3HP) biosynthetic pathway using a set of regulated promoters, they suppressed 3HP production during the glucose‐rich phase and activated it during glucose limiting. Specifically, regulation via the ICL1 promoter resulted in a 1.7‐fold increase in 3HP titer compared to constitutive expression driven by the PGK1 promoter.^[^ ^104^ ^]^

### 3.3 Dynamic Regulation by Quorum‐Sensing

In microbial communities, quorum sensing (QS) mechanisms influence the balance between growth and product synthesis by coordinating cellular behavior in response to population density.^[^ ^105^ ^]^ Pathway‐independent QS‐based dynamic regulation has emerged as a fundamental and widely adopted tool for fine‐tuning gene expression in response to changes in cell density, eliminating the need for costly or toxic inducers.^[^ ^106^ ^]^ Important QS‐based strategies in metabolic engineering include the direct use of natural QS systems, integration of QS with other regulatory tools, regulation of microbial cocultures, and the application of synthetic or non‐natural QS systems that respond to endogenous signals.^[^ ^107^ ^]^ These strategies enable dynamic switching between biomass accumulation and product formation, enhancing both productivity and efficiency.

The EsaI/EsaR QS system from *Pantoea stewartia* is a well‐characterized intercellular receptor (I/R) model in Gram‐negative bacteria.^[^ ^108^, ^109^ ^]^ Applied as a pathway‐independent genetic control module, this system was used in *E. coli* to dynamically reallocate glycolytic flux toward engineered heterologous pathways. As a result, myo‐inositol titers increased 5.5‐fold, and glucaric acid production improved from undetectable levels to over 0.8 g L^−1^ substantially outperforming parent strains lacking dynamic flux control.^[^ ^22^ ^]^ Beyond regulating gene expression at specific cell densities, modular and bifunctional QS systems offer powerful control over pathway activity. The Phr60‐Rap60‐Spo0A system was used to dynamically balance *B. subtilis* growth with menaquinone‐7 (MK‐7) biosynthesis, achieving a 40‐fold increase in MK‐7 production, from 9 to 360 mg L^−1^ in shake flasks.^[^ ^110^ ^]^ Similarly, bifunctional switches based on the Esa QS system were applied in *E. coli* for the production of poly‐β‐hydroxybutyrate (PHB) and 5‐aminolevulinic acid (ALA), resulting in 6‐ and 12‐fold increases, respectively.^[^ ^111^ ^]^ A modular PhrQ‐RapQ‐ComA QS system was employed in *B. subtilis* 168 to dynamically regulate MK‐4 biosynthesis, effectively balancing growth and production.^[^ ^112^ ^]^ This strategy increased MK‐4 titers from 120.1 ± 0.6 to 178.9 ± 2.8 mg L^−1^ in shake flasks. The *lux* QS system from *Vibrio fischeri*, one of the most extensively studied QS systems, serves as a tunable cell density sensor‐regulator.^[^ ^113^, ^114^ ^]^ It enables auto‐induction of target gene expression in response to QS signals, with the threshold cell density adjustable via the concentration of an external inducer. This capability allows metabolic flux to be redirected from central carbon metabolism toward synthetic isopropanol production once the desired cell density is reached, leading to a substantial increase in isopropanol titers.^[^ ^115^ ^]^ Doong et al. (2018) employed a layered dynamic regulation system in *E. coli* K‐12 to significantly enhance D‐glucaric acid production.^[^ ^116^ ^]^ They integrated a pathway‐independent quorum‐sensing circuit to downregulate glycolysis and shift cells from growth to production mode, alongside a myo‐inositol‐responsive module that activates downstream enzyme expression upon intermediate buildup. This dual‐regulation strategy yielded the highest glucaric acid titers reported for *E. coli* K‐12.^[^ ^116^ ^]^ Collectively, dynamic metabolic regulation addresses pathway challenges like toxic intermediates, competing reactions, and flux imbalances. By implementing pathway‐specific systems‐such as metabolite‐sensing biosensors‐and pathway‐independent systems like quorum‐sensing circuits, dynamic control enables cells to autonomously shift from growth to production modes, thereby balancing cellular health with high product yield and mitigating metabolic stress.^[^ ^51^ ^]^

## 4 Orthogonal System Design to Insulate Cell Growth and Product Synthesis

Orthogonal design represents a pivotal strategy for addressing the challenge of balancing cellular growth and product synthesis in microbial production systems. By incorporating orthogonal circuits or pathways that operate independently of the host's native metabolic processes, this approach effectively decouples growth from production, allowing the two processes to occur either simultaneously or in a controlled, optimized sequence.^[^ ^117^ ^]^ This design strategy enhances both metabolic flexibility and efficiency, while reducing competition for cellular resources between biomass formation and product biosynthesis. Orthogonal modules such as catabolic division or orthogonal translation can be engineered to utilize distinct enzymes, substrates, or codons that do not interfere with the host's core metabolic functions (Figure 1C). This separation enables the host organism to carry out essential processes, including energy metabolism, replication, and growth, without compromising the efficient synthesis of target compounds. By decoupling these functional pathways or synthetic components, orthogonal design maximizes overall metabolic efficiency and supports the simultaneous execution of native cellular activities and high‐level product formation.

### 4.1 Catabolic Division of Mixed Carbon Sources

The catabolic division of mixed carbon sources refers to a complex metabolic process in which microorganisms or cells degrade multiple carbon substrates either simultaneously or sequentially to generate energy and biomass.^[^ ^118^ ^]^ This metabolic flexibility is essential in natural environments, where organisms are often exposed to a variety of potential nutrient sources. Efficient utilization of mixed carbon substrates involves sophisticated regulatory mechanisms that allow cells to prioritize certain substrates over others‐commonly through carbon catabolite repression (CCR).^[^ ^119^ ^]^ A deep understanding of catabolic pathways and their regulatory control in mixed carbon environments is critical for advancing applications in biotechnology, waste valorization, and industrial fermentation. By leveraging these metabolic processes, researchers can optimize microbial growth and product biosynthesis, thereby improving the overall efficiency of bio‐based production systems. Numerous studies on co‐utilization of carbon sources have focused on synthesizing additive precursors or engineering co‐culture processes. For instance, co‐production of caffeic acid from xylose and glucose has been demonstrated.^[^ ^120^ ^]^ Chlorogenic acid has been co‐produced using a phenylalanine‐deficient MG09 strain (which converts xylose into caffeic acid), and a tyrosine‐deficient BD07 strain (which converts glucose into quinic acid).^[^ ^121^ ^]^ Additionally, the synergistic metabolism of glucose and formate has been shown to enhance short‐chain organic acid yields in *E. coli*.^[^ ^122^ ^]^ Despite these advances, achieving the appropriate distribution of carbon flux between cell growth and product formation remains a major challenge for high‐level biochemical production. Using mixed carbon substrates that feed into distinct metabolic pathways provides a rational framework for dividing cellular metabolism into separate growth and production modules. An et al. (2021) summarized strategies for co‐utilizing multiple carbon sources–such as glucose, xylose, arabinose, glycerol, and C1 compounds–offering valuable insights into pathway integration and flux control.^[^ ^123^ ^]^ Ma et al. (2024) review further reviewed strategies for co‐utilization, including alleviating of CCR, engineering of secondary carbon source transport and metabolism, enforcement of co‐utilization in monocultures, co‐culture‐based approaches, and evolutionary adaptation techniques.^[^ ^124^ ^]^ Recent developments in the catabolic division of mixed carbon sources as a strategy to balance biomass formation and product biosynthesis in microbial systems are described below.

**Figure 3:**
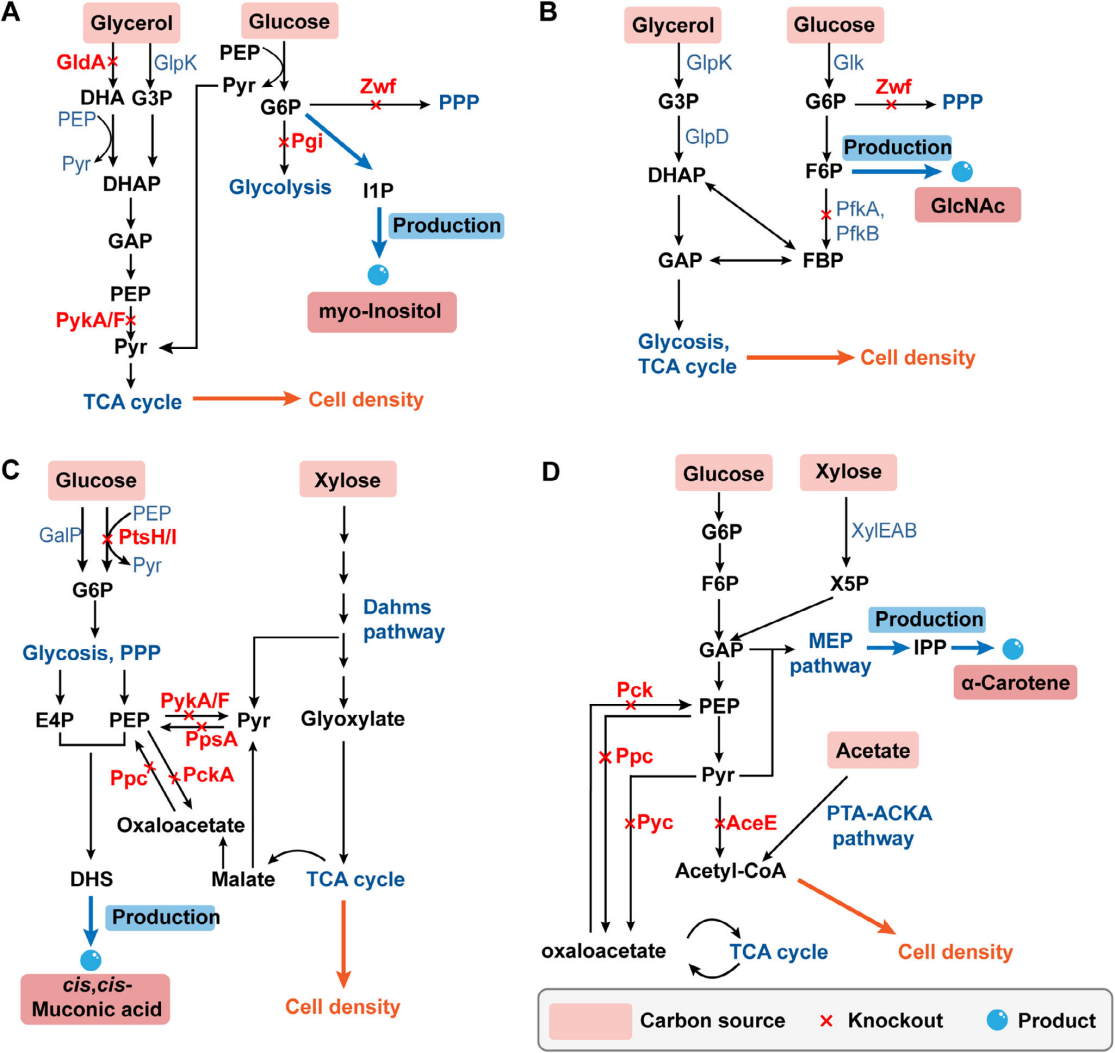
Catabolic division of labor in the utilization of mixed carbon sources. A) Schematic representation of myo‐inostitol production via the synergetic carbon utilization and Pyr‐coupled mechanism in *E. coli*. G6P is the metabolic node of this study. Adapted with permission.^[^ ^126^ ^]^ Copyright 2020, Wiley. B) Strategy for the catabolic division of labor in the utilization of mixed glycerol/glucose carbon sources for GlcNAc production. Adapted with permission.^[^ ^127^ ^]^ Copyright 2021, American Chemical Society. C) Metabolic design of the strain in which the Dahms pathway was introduced for cell density. Adapted with permission.^[^ ^129^ ^]^ Copyright 2021, Springer Nature. D) Schematic diagram illustrating the engineered partitioning of carbon‐source flux between glycolysis and the TCA cycle to facilitate α‐carotene biosynthesis. Adapted with permission.^[^ ^20^ ^]^ Copyright 2024, Elsevier. I1P, myo‐inositol 1‐phosphate; DHS, dehydroshikimate. GlcNAC, N‐Acetyl‐glucosamine. MEP, Methylerythritol phosphate, IPP: Isopentenyl diphosphate. Red font shows the deletion of the key enzymes. The metabolites in the green boxes represent the carbon sources. Red crosses indicate the disruption of metabolic pathways.

In nature, glucose is a common and preferred carbon and energy source for catabolic processes and was therefore selected as the primary carbon source in many metabolic engineering strategies.^[^ ^125^ ^]^ Co‐utilization typically involves glucose in combination with other carbon sources such as glycerol, xylose, arabinose, and others. A well‐known example of synergistic glucose and glycerol utilization illustrates the principle of metabolic coupling.^[^ ^126^ ^]^ In the biosynthesis of myo‐inositol (MI), G6P is a critical precursor, and its availability directly influences product yield (**Figure** **3**A). In one study, glycerol was metabolized to support cell growth, while glucose was preserved as a precursor for MI synthesis. This was achieved by blocking glycolysis and the PPP through deletion of the *zwf* and *pgi* genes. Growth and production were coupled via the phosphotransferase system (PTS), with pyruvate serving as the linking metabolite (Figure 3A). The final engineered strain achieved high MI titer and yield, producing 76 g L^−1^ in fed‐batch cultivation, thereby demonstrating strong scale‐up potential. This study exemplifies the integration of a pyruvate‐centric coupling strategy with mixed carbon sources utilization to significantly enhance product titer. F6P is another key precursor, especially relevant for the efficient production of N‑acetyl‐glucosamine (GlcNAc). To redirect carbon flux, the *pfkA* gene was deleted, and glycerol utilization was enhanced by introducing a mutant *glpK* gene.^[^ ^127^ ^]^ This strategy enabled glycerol to primarily support cell growth and maintenance, conserving glucose for GlcNAc biosynthesis (Figure 3B). The resulting strain, GLALD‐7, produced 179.7 g L^−1^ of GlcNAc using a mixed carbon source of glycerol and glucose at a 1:8 mass ratio in a 5 L bioreactor. A similar synergistic strategy for glucose and glycerol co‐utilization was applied to *Komagataella phaffii*, a methylotrophic yeast traditionally known for metabolizing methanol as its sole carbon source.^[^ ^128^ ^]^ In this approach, glycerol was used to support cell growth while glucose was reserved for product biosynthesis. This effectively divided cellular metabolism into distinct growth and production modules, establishing a flexible and scalable fermentation platform for biochemical production.^[^ ^128^ ^]^

In addition to using a glycerol‐glucose mixture, combining glucose with other carbon sources such as xylose or arabinose can also be effective in enhancing bioproduct yield. For example, deletion of *pgi* and *zwf* eliminates catabolite repression and prevents *E. coli* from utilizing glucose for biomass formation.^[^ ^130^ ^]^ Supplementation with _L_‐arabinose, glycerol, and _D_‐xylose supports cell growth and increases glucaric acid production by 9‐ to 18‐fold compared to using glucose as the sole carbon source in the Δ*pgi* Δ*zwf* strain. A strategy known as parallel metabolic pathway engineering (PMPE) was developed to produce shikimate pathway derivatives using a glucose‐xylose co‐substrate system.^[^ ^129^ ^]^ In this approach, glucose is primarily directed toward the synthesis of the target compound, while xylose supplies essential metabolites to support cell growth (Figure 3C). Using PMPE, engineered *E. coli* strains produced 4.09 g L^−1^ of *cis*, *cis*‐muconic acid with a high yield (0.31 g g^−1^ of glucose) and achieved L‐tyrosine production at 64% of the theoretical yield.^[^ ^129^ ^]^

A combination of glucose, xylose, and acetate can be used to enhance α‐carotene production in a high‐efficiency *C. glutamicum* cell factory.^[^ ^20^ ^]^ To prevent carbon flux glycolysis and the PPP from the tricarboxylic acid (TCA) cycle, key metabolic connections were disrupted by deleting the *pck, pyc*, *ppc*, and *aceE* genes (Figure 3D). Acetate was then introduced as a third carbon source, feeding directly into the TCA cycle without interfering with glycolysis or the PPP. This strategy allowed glucose and xylose‐processed through a heterologous xylose pathway to be primarily directed toward α‐carotene biosynthesis, while acetate exclusively supported cell growth by supplying TCA cycle intermediates.^[^ ^20^ ^]^

In summary, the strategies discussed highlight the importance of optimizing carbon source allocation across key metabolic pathways, including glycolysis, the PPP, the MEP pathway, and others. These approaches aim to alleviate CCR while simultaneously enhancing the uptake and metabolism of diverse carbon sources within the cellular environment. By strategically reallocating carbon flux, these methodologies improve metabolic efficiency and product yield, fostering a more robust and adaptable cellular metabolism capable of effectively utilizing a broader range of substrates. This optimization not only helps to overcome the constraints imposed by CCR but also enhances the organism's overall metabolic versatility and productivity. By redirecting metabolic fluxes, this engineering strategy enables cells to efficiently co‐utilize multiple carbon sources without the typical inhibitory effects associated with CCR. Ultimately, the goal of carbon source division is to engineer resilient microbial strains with enhanced metabolic flexibility‐capable of thriving in complex environments and converting mixed carbon substrates into valuable biochemicals or biofuels‐thereby improving the overall yield and efficiency of industrial bioprocesses.

### 4.2 Genetic Codon Expansion to Balance Growth and Synthesis

In nature, organisms use a highly conserved codon table to encode 20 canonical amino acids, which serve as the fundamental building blocks of protein synthesis.^[^ ^131^ ^]^ Throughout evolution, protein structure and function have been diversified by random mutations that alter amino acid sequences; however, this process lacks precision and control. Genetic codon expansion (GCE), enabled by the development of orthogonal translation systems, offers a powerful strategy to precisely manipulate protein structure and function.^[^ ^132^ ^]^ In the context of metabolic engineering, GCE can be applied to balance cellular growth and product biosynthesis. By incorporating non‐canonical amino acids (ncAAs) or optimizing codon usage, this approach allows for fine‐tuned control of protein synthesis,^[^ ^133^, ^134^ ^]^ thereby reducing competition between biomass formation and the biosynthesis of target compounds. This precise allocation of translational resources has significant potential to enhance the efficiency of industrial bioprocesses by optimizing metabolic flux and improving the coordination between cellular proliferation and production.

Aminoacyl‐tRNA synthetase (aaRS)/tRNA pairs play a central role in translation.^[^ ^135^, ^136^ ^]^ Ensuring orthogonality‐meaning these engineered pairs do not cross‐react with native cellular machinery‐is critical for the functionality of GCE systems and their downstream applications. By introducing orthogonal aaRS/tRNA pairs into host organisms and encoding non‐canonical codons into target genes, specific ncAAs can be incorporated into proteins during translation.^[^ ^136^ ^]^ GCE‐based orthogonal translation systems accomplish this by reassigning non‐standard codons(e.g., the amber stop codon) to ncAAs, allowing precise metabolic control and novel protein functions. Amber stop codon suppression has become a routine method for site‐specific incorporation of ncAAs into proteins, significantly broadening the functional scope of engineered proteins.^[^ ^132^ ^]^ For instance, Casas et al. (2020) demonstrated that a growth‐decoupled BL21(DE3) gp2 *E. coli* strain can enhance the expression of synthetic ncAA‐labeled proteins using orthogonal aminoacyl‐tRNA synthetase/suppressor tRNA systems.^[^ ^137^ ^]^ Remarkably, depending on the protein target, expression levels comparable to those of wild‐type protein were achieved, even under fed‐batch fermentation conditions.

Orthogonal translation systems enable the precise regulation of target protein expression while preventing interference from the host's endogenous metabolic activities. For the efficient bioproduction of N‐acetylglucosamine and N‐acetylneuraminic acid, a strategy known as GCE‐based Cell Growth and Biosynthesis Balance Engineering (GCE‐CGBBE) has been developed.^[^ ^24^ ^]^ This approach controls the expression of genes involved in both cell growth and metabolic flux by establishing ncAA‐dependent expression patterns. Using the GCE‐CGBBE strategy in genetically recoded *E. coli* Δ321AM, glycolysis and N‐acetylglucosamine biosynthesis were precisely balanced through controlled addition of the ncAA pAcF,^[^ ^138^ ^]^ resulting in a 4.54‐fold increase in titer (12.77 g L^−1^).^[^ ^24^ ^]^ Additionally, the GCE‐CGBBE system was successfully applied to non‐recoded *B. subtilis* by incorporating amber stop codons into key genes regulating metabolic flux and growth. The addition of the ncAA OMeY led to a 2.34‐fold increase in titer (4.72 g L^−1^).^[^ ^24^ ^]^ Notably, this strategy is pathway‐independent and utilizes ncAAs not naturally found in standard media or growth environments, minimizing background interference and enhancing system control.

The GCE‐based orthogonal translation system thus represents a powerful tool for balancing cellular growth and product formation, particularly in metabolic engineering. By reassigning non‐canonical codons to ncAAs, this technology allows for precise, programmable control of protein synthesis‐extending functional capabilities beyond those accessible through natural evolution and reducing competition between growth and biosynthesis. As demonstrated in both *E. coli* and *B. subtilis*, the GCE‐CGBBE approach significantly enhances production yields and offers a versatile, modular platform for optimizing diverse bioprocesses. Its adaptability across different organisms and pathways underscores its potential for broader industrial applications. However, several challenges remain. These include improving the efficiency of ncAA incorporation, minimizing potential effects on protein folding, and ensuring robust orthogonality to prevent unintended interactions with host cellular machinery. Future research may focus on expanding the range of usable ncAAs, enhancing the performance of engineered aaRS/tRNA pairs, and optimizing the scalability of these systems for industrial bioproduction. Ultimately, the flexibility and precision of GCE‐based strategies position them as transformative tools for improving the efficiency, control, and versatility of biotechnological applications across a wide range of fields.

## 5 Microbial Consortia Engineering to Regulate Growth and Production

Microorganisms naturally exist within complex consortia, engaging in dynamic interactions and forming diverse communities, such as bacterial‐bacterial, bacterial‐fungal, and fungal‐fungal associations.^[^ ^139^ ^]^ Microbial co‐culturing seeks to replicate and harness these interactions by creating synthetic communities that mimic natural ecosystems. In the context of metabolic engineering, microbial co‐culturing consortia have emerged as a promising strategy to balance cellular growth and product synthesis. Unlike monocultures, microbial consortia exploit the complementary metabolic capabilities of different species or strains, allowing for the division of labor among community members.^[^ ^140^ ^]^ This division reduces the metabolic burden on individual cells and enables the processing of complex substrates or metabolic pathways that would be difficult for a single organism to manage alone (Figure 1D).^[^ ^141^ ^]^ By precisely engineering interspecies interactions and optimizing resource distribution, microbial consortia can achieve dynamic regulation of growth and production, leading to improved yields and enhanced process stability. These advantages make co‐culturing systems an increasingly valuable tool for the biosynthesis of a wide range of chemicals, fuels, and pharmaceuticals.

Microbial cooperative interactions play a pivotal role in the design and optimization of synthetic microbial consortia, enabling co‐cultivation strategies that promote balanced cell growth and enhance the production of valuable natural metabolites.^[^ ^142^ ^]^ In such systems, one strain (e.g., Strain A) may produce enzymes or metabolites that are utilized by another strain (e.g., Strain B), thereby supporting its growth and facilitating the synthesis of a desired product. This cooperative design in metabolic engineering focuses on optimizing resource utilization, reducing metabolic burden, improving product selectivity and yield, expanding metabolic capabilities, and enhancing flexibility and sustainability throughout the production process.

Microbial co‐cultures composed of metabolically engineered strains‐whether from the same species or different species‐have emerged as a promising approach to harness interspecies interactions and optimize complex metabolic pathways for diverse biotechnological applications.^[^ ^143^ ^]^ For example, Gao et al. (2023) demonstrated a cooperative consortium in which *C. glutamicum* produced proline to support *B. subtilis* growth, while *B. subtilis* contributed to the high‐level production of fengycin.^[^ ^144^ ^]^ Rong et al. (2024) engineered a xylitol‐producing *E. coli* strain utilizing CRISPRi‐mediated gene silencing to shift metabolism from aerobic to anaerobic conditions, effectively decoupling growth from production and improving yield.^[^ ^145^ ^]^ This strain also produces acetate as a byproduct, which was then utilized by a second *E. coli* strain–designed through constraint–based metabolic modeling‐to co‐consume glucose and acetate for the synthesis of a secondary metabolite.^[^ ^145^ ^]^ This approach, similar to that illustrated in Figure 2D, demonstrates how a consortium can facilitate the equivalent of “two fermentations in one go,” improving overall system efficiency. Co‐cultured microorganisms can also improve product titers by facilitating the removal or conversion of toxic byproducts during fermentation, thereby alleviating growth inhibition and maintaining metabolic balance.^[^ ^146^ ^]^ For example, in *Lactococcus lactis* subsp. *lactis* cultures, the accumulation of lactic acid inhibits both cell growth and nisin production. To address this, *Yarrowia lipolytica*—a yeast capable of utilizing lactate as an alternative carbon source—was introduced into the co‐culture. This intervention reduced lactic acid levels by 10%, promoted *L. lactis* growth, and enhanced nisin production by 50% compared to monoculture conditions.^[^ ^147^ ^]^ In another example, Kang et al. (2022) developed a “Population Guider” genetic circuit in *E. coli* that degrades ampicillin in response to 3‐HP production.^[^ ^148^ ^]^ When co‐cultured with alginate‐utilizing *Vibrio* sp. Dhg under ampicillin selection pressure, this system enabled population balance and cooperation, resulting in a 4.3‐fold increase in 3‐HP yield over 48 hours compared to a conventional co‐culture approaches.^[^ ^148^ ^]^ These examples underscore the power of cooperative microbial consortia to improve bioproduction outcomes by enabling dynamic metabolic interactions, optimizing resource distribution, and enhancing system robustness and scalability.

The studies discussed above demonstrate that microbial co‐culturing not only replicates the dynamics of natural microbial consortia but also offers substantial advantages in metabolic engineering. By harnessing the complementary metabolic capabilities of different species, synthetic consortia can distribute the metabolic burden, enhance resource utilization, and facilitate the conversion of complex substrates tasks that are often difficult to achieve with monocultures. Co‐culturing strategies have been shown to support balanced cell growth while simultaneously driving the production of valuable metabolites.^[^ ^146^ ^]^ This is particularly evident in systems where one strain produces essential enzymes or substrates that benefit another, leading to improved product yields and enhanced process stability. Despite these advantages, several challenges remain. Precisely controlling interspecies interactions, maintaining community stability over extended fermentation periods, and scaling these systems for industrial use require further innovation. Future research should focus on the development of advanced computational modeling, synthetic biology tools, and adaptive process control strategies to optimize microbial consortia and fully realize their biotechnological potential.

## 6 Fermentation Process Control to Balance Biomass Formation and Production

Fermentation control plays a critical role in balancing product synthesis and cell growth by fine‐tuning environmental and regulatory conditions throughout the fermentation bioprocess.^[^ ^54^, ^149^ ^]^ By manipulating key factors such as pH, temperature, nutrient availability, and DO levels, metabolic pathways can be optimized toward the desired product formation without compromising cell viability (Figure 1E).^[^ ^150^, ^151^, ^152^ ^]^ In addition, fermentation control enables dynamic adjustments during different growth phases, ensuring efficient resource allocation between biomass accumulation and product biosynthesis ^[^ ^153^, ^154^ ^]^ This approach helps to improve yields, maintain cell health and stability, and enhance the overall efficiency of industrial‐scale bioprocesses. When high specific growth rates and high production rates are incompatible, the maximum principle can be applied to determine the optimal strategy for maximizing product titers within a defined time frame.^[^ ^56^ ^]^ For strains engineered for high specific production rates, metabolic switching in a two‐stage fermentation process‐regulated by parameters such as temperature, DO, pH, and chemical inducers–offers an effective strategy to decouple growth from production and optimize both phases of the process.

**Figure 4:**
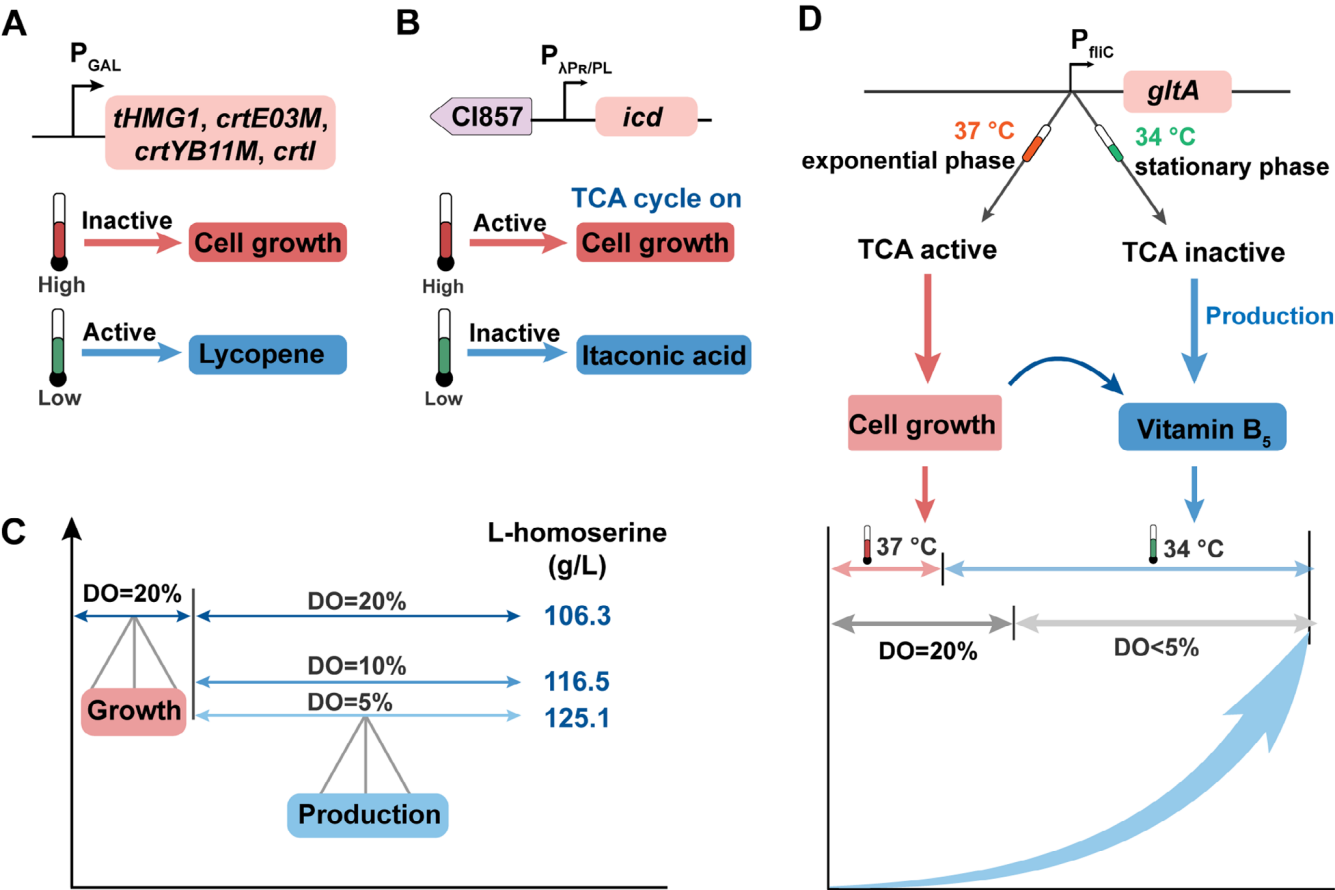
Fermentation process control to balance biomass formation and production. A) Gal4‐based temperature‐responsive regulatory strategy. The Gal4 mutant with temperature‐sensitive (TS) phenotype delivers transcriptional regulation to the P*_GAL_*‐controlled pathway genes. The proteins encoded by *tHMG1*, *crtE03M*, *crtYB11M*, and *crtI* genes catalyze the production of lycopene from HMG‐CoA (hydroxymethylglutaryl‐CoA). Adapted with permission.^[^ ^155^ ^]^ Copyright 2018, Wiley. B) Dynamic control of *icd* expression to regulate TCA cycle. At 37 °C, the repressor CI857 is inactive allowing *icd* expression for an active TCA cycle and cell growth. At 30 °C, the repressor becomes active and blocks transcription of *icd* by binding to the promoters pR/pL, and thus the TCA cycle redirecting flux to itaconic acid. Adapted with permission.^[^ ^66^ ^]^ Copyright 2018, Wiley. C) Effects of various DO control conditions including 20, 10, and 5% on L‐homoserine fermentation. Adapted with permission.^[^ ^160^ ^]^ Copyright 2024, American Chemical Society. D) Dynamically regulated *gltA* by GPP, temperature, and DO feedback control strategy to enhance vitamin B_5_ production. Temp, temperature. Adapted with permission.^[^ ^161^ ^]^ Copyright 2024, Elsevier.

In bioprocess engineering, the ability to decouple cell growth from product synthesis is essential for optimizing fermentation processes and maximizing product yields. Temperature‐sensitive genetic switches provide a powerful means to dynamically regulate metabolic pathways in response to environmental cues, allowing precise control over distinct growth and production phases. For example, a temperature‐sensitive Gal4 mutant, Gal4M9, was developed through directed evolution and employed as a protein switch in *ΔGAL80* yeast strain to regulate lycopene production (Figure 4A).^[^ ^155^ ^]^ During the initial growth phase, the strain proliferated rapidly and entered mid‐log phase, with lycopene levels remaining undetectable after 38 h. A temperature shift to 24 °C was then applied to activate lycopene biosynthesis. From 48 h onward, lycopene began to accumulate significantly, reaching 1.14 g L^−1^ by the end of fermentation at 200 h.^[^ ^155^ ^]^ However, sustaining lycopene production required maintaining the fermentation temperature at 24 °C throughout the production phase, resulting in high energy consumption. To overcome this limitation, a cold‐shock‐triggered temperature control system was developed by placing the wild‐type Gal4 under the control of Gal4M9. This system simultaneously controlled both the expression and activity of the transcriptional activator upon temperature shift and ensured continued Gal4 expression after reverting the temperature to 30 °C. This design compensated for the reduced activity of Gal4M9, allowing P*_GAL_*‐driven biosynthetic pathways to remain active without the need for prolonged low‐temperature fermentation. As a result, 320 mg L^−1^ tocotrienols were produced using a protocol of 30 °C for 40 h, 24 °C for 5 h (cold shock), followed by 30 °C until the end of the fermentation.^[^ ^156^ ^]^ Similarly, bacteriophage λ promoters (pR, pL) have been used in conjunction with the temperature‐sensitive repressor CI857, which is active at 30 °C and inactivated at 37 °C.^[^ ^157^, ^158^ ^]^ This temperature‐responsive system enables dynamic gene expression regulation. For instance, rather than simply inducing gene expression, temperature‐dependent expression of the *icd* gene (encoding isocitrate dehydrogenase) was used to control the TCA cycle and growth rate. High TCA cycle activity was maintained during the growth phase, while reduced TCA flux in the production phase allowed for enhanced itaconic acid biosynthesis. In a study by Harder et al. (2018), this two‐stage temperature‐controlled strategy led to a 22% increase in overall productivity and a 48% increase in peak productivity compared to a one‐stage process (**Figure** **4**B).^[^ ^66^ ^]^ Additionally, a thermo‐controllable genetic switch regulating *ldhA* expression was used to decouple growth from production in *E. coli* strain B0013‐070B, simplifying D‐lactate fermentation. This approach resulted in the production of 122.8 g L^−1^ of D‐lactate.^[^ ^159^ ^]^ Collectively, these studies highlight the potential of temperature‐sensitive genetic switches to decouple growth and production phases, enabling more efficient and flexible bioprocesses. By reducing energy consumption and improving yields, these systems offer a promising route to optimizing industrial‐scale fermentation.

DO levels act as a critical “switch” in the dynamic regulation of the TCA cycle. Attenuation or suppression of TCA cycle activity can effectively promote the production of chemicals that use pyruvate as a precursor.^[^ ^162^ ^]^ Fluctuations in DO levels influence the activity of transcription factors, thereby inducing shifts in metabolic fluxes.^[^ ^163^ ^]^ For the efficient production of L‐homoserine, a two‐stage DO‐controlled fermentation was implemented in a 5 L bioreactor. Initially, the DO level was maintained at approximately 20% to support cell growth until the culture reached an OD_600_ of 30. Subsequently, the DO was sequentially reduced to 20, 10, and finally 5% to redirect carbon flux from biomass formation to L‐homoserine biosynthesis. This strategy resulted in an L‐homoserine titer of 125.1 g L^−1^ within 60 h, with the most pronounced increase observed at 5% DO (Figure 4C).^[^ ^160^ ^]^ Similarly, in the development of a high‐yield *E. coli* strain for vitamin B_5_ [D‐pantothenic acid (D‐PA)] production, dynamic regulation via DO level, temperature shifts and GPP control was employed (Figure 4D).^[^ ^161^ ^]^ In this process, DO was maintained at 20% for the first 24 h and reduced to below 5% from 24 to 60 h. Simultaneously, the fermentation temperature was held at 37 °C from 0 to 12 h and lowered to below 34 °C for the remaining duration. These conditions yielded a final D‐PA titer of 83.26 g L^−1^ in a 5 L bioreactor, underscoring the system's potential for industrial production. Together, these studies demonstrate that dynamic regulation of metabolic pathways through environmental factors variables‐such as DO and temperature‐is an effective strategy to enhance chemical production in microbial fermentation. By integrating environmental control into metabolic engineering, it is possible to achieve higher titers, improve resource allocation, and boost overall process efficiency.

Acidic pH conditions are generally favorable for organic acid production, although low pH can also inhibit bacterial growth.^[^ ^164^ ^]^ Ernst et al. (2024) reported that a hyper‐producing strain could achieve the theoretical maximum itaconate yield at pH 3.6 during the production phase, although this required substantial base addition.^[^ ^165^ ^]^ Lowering the pH further to 2.8 significantly reduces base consumption but negatively impacted yield, titer, and production rate, and caused growth defects. When the pH was adjusted to 5.0 and the feeding phase was extended, the final itaconic acid titer reached 125.2 ± 14.6 g L^−1^—one of the highest titers reported for Ustilaginaceae fungi using NaOH titration. Although pH was not actively controlled in that study, it clearly demonstrated that pH can significantly influence both cell growth and product formation in itaconate production systems.

Decoupling cell growth from product formation allows enables rapid biomass accumulation without the inhibitory effects of product synthesis. This can be achieved using chemical inducers such as isopropyl β‐D‐thiogalactoside (IPTG).^[^ ^166^, ^167^, ^168^, ^169^ ^]^ Although IPTG‐inducible promoters are widely used for recombinant protein expression, they are less suited for industrial applications due to the high cost of IPTG, its potential toxicity to cells, and operational limitations such as the need for accurate cell density monitoring and timing of induction.^[^ ^27^, ^170^ ^]^ Cho et al. (2024) reported that lowering the IPTG concentration to 10 µm alleviated growth inhibition, and omitting IPTG altogether‐allowing only basal‐level gene expression‐improved 2,6‐pyridinedicarboxylic acid (2,6‐PDCA) production with minimal impact on growth.^[^ ^171^ ^]^ In addition, precise modulation of the substrate feed rate enables dynamic control of the specific growth rate (µ), allowing the coordination of biomass formation with biosynthetic flux by adjusting nutrient availability.^[^ ^172^ ^]^ For example, Kim et al. (2024) showed that manual adjustment of the carbon source feed rate to maintain concentrations between 5 and 15 g L^−1^ optimized both cell growth and succinic acid production.^[^ ^173^ ^]^ These findings collectively highlight the importance of environmental and regulatory controls‐including pH, inducers, and feed strategies‐for decoupling growth from production and enhancing overall bioprocess performance.

## 7 Metabolic Modeling to Harmonize Cell Growth and Product Synthesis

Achieving high titer, rate, and yield (TRY) while ensuring scalability in microbial production remains a significant challenge, primarily due to the inherent trade‐off between carbon allocating for biomass growth versus product synthesis.^[^ ^174^ ^]^ Computational modeling of metabolic networks has become a standard approach in the rational design of production strains for metabolic engineering.^[^ ^175^ ^]^ A foundational strategy in this context is the stoichiometric coupling of biomass formation and product synthesis, ensuring that the biosynthesis of the target compound is obligatorily linked to and thereby drives cellular growth. Over the past decade, this approach has become a cornerstone strategy of metabolic engineering and computational strain design.^[^ ^38^ ^]^ The core concept is to embed product formation within essential metabolic processes, effectively making the synthesis of the desired metabolite a necessary “by‐product” of cell proliferation.^[^ ^176^ ^]^ By leveraging growth‐product flux coupling, target compound biosynthesis becomes indispensable to central biomass‐forming pathways. As a result, any disruption of the engineered circuit incurs a fitness penalty, thereby providing evolutionary stability and selection pressure that favors retention of the production trait. This intrinsic coupling not only safeguards engineered pathways but also streamlines strain optimization. Furthermore, adaptive laboratory evolution (ALE) can be employed to refine cellular metabolism, relieve metabolic bottlenecks, and enhance production performance ^[^ ^177^, ^178^, ^179^ ^]^ When combined with growth‐coupled design principles, ALE serves as a powerful tool to amplify production outputs and improve strain robustness, paving the way for scalable and economically viable bioprocesses.

Several computational methods have been developed to predict the necessary reaction deletions and/or insertions for designing growth‐coupled strains, including OptKnock,^[^ ^180^ ^]^ RobustKnock,^[^ ^181^ ^]^ modCell2,^[^ ^182^ ^]^ OptCouple,^[^ ^183^ ^]^ and StrainDesign ^[^ ^184^ ^]^, among others (Figure 1F). OptKnock pioneered a bi‐level mixed‐integer linear programming (MILP) framework to identify gene deletion strategies in *E. coli*.^[^ ^180^ ^]^ By enforcing stoichiometric constraints, the model redirects carbon, redox, and energy fluxes toward both biomass formation and target‐product synthesis. It's in silico predictions for succinate, lactate, and 1,3‐propanediol overproduction have shown strong agreement with experimentally observed mutant phenotypes.^[^ ^180^ ^]^ However, growth coupling is not universally feasible for all metabolites. Achieving strong coupling–where target‐metabolite production is essential in all viable steady‐state flux distributions, even under suboptimal growth conditions–makes product formation an inescapable requirement of the organism's metabolism.^[^ ^37^, ^38^, ^185^ ^]^ Nevertheless, von Kamp and Klamt (2017) demonstrated that, under appropriate conditions, growth‐production coupling is theoretically feasible for nearly all metabolites in genome‐scale models of five major production hosts: *E. coli*, *S. cerevisiae*, *C. glutamicum*, *A. niger*, and *Synechocystis* sp. PCC 6803.^[^ ^38^ ^]^ These organisms span both prokaryotic and eukaryotic domains, as well as heterotrophic and photoautotrophic lifestyles, underscoring the broad applicability of growth coupling as a universal strain design principle.^[^ ^38^ ^]^ In a compelling application, Banerjee et al. (2020) employed a minimal cut set (MCS) approach to predict and delete specific metabolic reactions, thereby enforcing robust growth coupling for the biosynthesis of indigoidine, a non‐native product.^[^ ^174^ ^]^ Using a genome‐scale metabolic model of *Pseudomonas putida* KT2440, the researchers computed 63 candidate MCS solution sets. They then applied multiplexed CRISPR interference (CRISPRi) to simultaneously implement 14 gene knockdowns in a single strain marking the first experimental realization of such an extensive MCS‐based design. This architecture shifted indigoidine production from the stationary to the exponential growth phase, resulting in a titer of 25.6 g L^−1^, productivity of 0.22 g L^−1^ h^−1^, and ≈50% of the maximum theoretical yield (0.33 g indigoidine g^−1^ glucose).^[^ ^174^ ^]^ Jensen et al. (2019) introduced OptCouple, a constraint‐based modeling framework that simultaneously identifies optimal combinations of gene knockouts, gene insertions, and medium supplements to achieve growth‐coupled production of a target compound.^[^ ^183^ ^]^ This method also employs a bi‐level programming approach to maximize the potential for growth‐production coupling, thereby guiding metabolic engineering strategies. StrainDesign, a Python extension of COBRApy, integrates several leading metabolic design algorithms‐including OptKnock,^[^ ^180^ ^]^ RobustKnock,^[^ ^181^ ^]^ OptCouple,^[^ ^183^ ^]^ and MCS ^[^ ^186^ ^]^‐within a unified, modular framework.^[^ ^184^ ^]^ This platform enables the systematic implementation of combined gene/reaction knockouts, pathway insertions, and regulatory interventions. By constraining all feasible flux distributions to satisfy both a minimum growth rate and a minimum product yield (as in OptEnvelope), the framework maintains cellular viability while enforcing target compound synthesis.^[^ ^187^ ^]^ This explicit delineation between acceptable and unacceptable flux distributions stoichiometric coupling between growth and production, enabling the design of robust growth‐coupled strains that meet predefined performance benchmarks.^[^ ^37^ ^]^ As a result, users gain precise control over the growth‐production trade‐off, allowing strain designs to be tailored for industrially relevant titers, rates, and yields without compromising essential biomass formation.

Despite these advances, significant challenges remain in translating in silico designs into industrially viable strains. Factors such as unmodeled regulatory constraints, the metabolic burden imposed by heterologous pathway elements, and the need for scalable and controllable fermentation conditions complicate the path from computational prediction to practical implementation. Future efforts will likely incorporate multi‐omics data integration, dynamic control circuits, and machine‐learning driven model refinement to better account for regulatory layers and non‐stoichiometric effects. Ultimately, coupling such enhanced computational models with ALE and high‐throughput holds great promises for bridging the gap between theoretical yield and realized TRY (titer, rate, yield), enabling the development of fully optimized microbial cell factories for next‐generation biomanufacturing.

## 8 Conclusion

Microbial production of fuels, chemicals, and materials from renewable resources is becoming increasingly important for reducing the carbon emissions.^[^ ^188^, ^189^, ^190^ ^]^ However, in many cases, the native metabolic pathways of host strains are insufficient for the efficient biosynthesis of target compounds.^[^ ^191^ ^]^ Modifying these pathways to enhance productivity can create conflicts between intracellular metabolism and product synthesis. In this paper, we reviewed recent advances aimed at balancing cell growth with product synthesis. We highlighted key strategies including pathway engineering, dynamic regulation, orthogonal design, microbial co‐culturing, fermentation process control, and metabolic modeling. These approaches offer valuable insights for regulating cellular metabolism and improving product bioproduction, providing useful references for future research.

Central carbon metabolism (CCM)–which includes glycolysis, TCA cycle, and the PPP–is fundamental to cellular growth and physiological activities.^[^ ^192^, ^193^ ^]^ Because product synthesis competes with CCM for cellular resources, balancing the two often requires attenuating, blocking, or finely regulating CCM to optimize resource allocation. Establishing an efficient microbial cell factory begins with a comprehensive understanding of the biosynthetic characteristics of the target product, including the identification of relevant enzymes, substrates, cofactors, and potential by‐products.^[^ ^194^ ^]^ For example, combining orthogonal design with pathway engineering can decouple cell growth from product synthesis. However, one persistent challenge is the establishment of an effective metabolic driving force. This driving force is essential for optimizing resource utilization, directing carbon flux toward the desired product while sustaining sufficient cell growth. As shown in Figure 3A, researchers identified pyruvate as a by‐product of glucose metabolism and constructed efficient myo‐inositol‐producing strains by coupling pyruvate‐driven growth with orthogonal pathways that utilize glucose and glycerol as mixed carbon sources.^[^ ^126^ ^]^ The translation of coding sequences into proteins based on codon recognition is fundamental to genome expression and cellular function.^[^ ^195^ ^]^ GCE refers to the reprogramming of the genetic code to include additional codons beyond the 64 naturally occurring ones.^[^ ^134^, ^196^ ^]^ This expansion enables cells to incorporate ncAAs into proteins, allowing for the synthesis of proteins with novel chemical properties or catalytic activities. GCE relies on reengineering the translation machinery‐particularly tRNA and aaRS‐to recognize new codons and selectively pair them with ncAAs.^[^ ^134^ ^]^ This orthogonal system prevents interference with native translation processes, enabling more precise and programmable control of protein expression.

Dynamic regulation using metabolic switches or QS systems is a powerful strategy for optimizing microbial production processes.^[^ ^17^ ^]^ These regulatory mechanisms enable cells to adjust their metabolic activities in response to environmental cues, internal metabolic states, or population density, thereby improving the efficiency of both growth and product synthesis. However, there approaches also present several challenges, including complexity, metabolic burden, signal interference, population dependency, feedback inhibition, and issues of genetic stability. Effective system design must take these factors into account to ensure robust and consistent performance, particularly in industrial applications.

Fermentation process optimization is another critical component of microbial biotechnology, focused on maximizing product yield and efficiency. It is a fundamental requirement for the successful commercialization of scientific and technological innovations. Achieving high yields in microbial fermentation is inherently complex and depends on the coordinated control of cellular growth and product biosynthesis. By understanding metabolic trade‐offs, implementing dynamic regulation systems, precisely controlling fermentation parameters (e.g., DO, temperature, and pH), and leveraging feedback mechanisms, researchers can develop highly efficient and sustainable bioproduction processes.

As the demand for bio‐based products increases, mastering the balance between growth and production will be essential for driving innovation and achieving economic viability in the biotechnology industry. Systems metabolic engineering‐which integrates systems biology, synthetic biology, and evolutionary engineering with traditional metabolic engineering‐has emerged as a key approach for developing high‐performance microbial strains.^[^ ^197^ ^]^ Systems biology enables a comprehensive understanding of cellular responses to genetic and environmental changes. By incorporating multi‐omics analyses‐such as genomics, transcriptomics, proteomics, and metabolomics‐researchers can uncover the complex regulatory networks that link growth and product formation. This holistic understanding is crucial for identifying metabolic bottlenecks and designing targeted interventions to improve flux distribution and efficiency. The potential of systems metabolic engineering to establish robust microbial cell factories hinges on its ability to balance product synthesis with essential cellular functions. By combining advanced modeling tools, synthetic biology techniques, and omics‐based insights, researchers can create optimized strains capable of high‐yield, sustainable production of valuable biochemical. Continued exploration of these strategies will drive progress in metabolic engineering and facilitate the successful implementation of microbial cell factories across a wide range of biotechnological applications.

## Conflict of Interest

The authors declare no conflict of interest.
